# Case report: A mesocolic lymphangioma in a 14-year-old child resected by laparoscopic surgery

**DOI:** 10.3389/fonc.2022.1034563

**Published:** 2022-11-09

**Authors:** Xuping Feng, Xinyang Chen, Qingbo Feng, Xiaoyin Liu, Hancong Li, Hao Chen, Zhaolun Cai, Jiaxin Li

**Affiliations:** ^1^ Department of Liver Surgery & Liver Transplantation, State Key Laboratory of Biotherapy and Cancer Center, West China Hospital, Sichuan University and Collaborative Innovation Center of Biotherapy, Chengdu, Sichuan, China; ^2^ Laboratory of Liver Surgery, West China Hospital, Sichuan University, Chengdu, Sichuan, China; ^3^ West China School of Medicine, West China Hospital, Sichuan University, Chengdu, Sichuan, China; ^4^ Department of Neurosurgery, West China Hospital, Sichuan University, Chengdu, Sichuan, China; ^5^ Gastric Cancer Center, West China Hospital, Sichuan University, Chengdu, Sichuan, China; ^6^ DaFang County People's Hospital, Bijie, Guizhou, China

**Keywords:** mesocolon, cystic lymphangioma, case report, laparoscopic surgery, literature review

## Abstract

**Introduction:**

Cystic lymphangioma is a benign malformation tumor of the lymphatic system. Its location is variable, and mesocolic localization remains extremely rare.

**Case presentation:**

We report a case of right mesocolon giant cystic lymphangioma in a previously healthy 14-year-old boy who was successfully managed through a minimally invasive laparoscopic excision. The patient presented with 8 months of dull abdominal pain, sporadic, located on the peri-umbilicus, exacerbated for a month. An abdominal computed tomography (CT) revealed a large, multiseptated cystic mass on the right mesocolon. Right mesocolic excision using a laparoscope was performed on this patient. He was discharged on the fifth day without complications. Recurrence was not detected in three months of follow-up.

**Conclusion:**

Cystic lymphangiomas in the mesocolon are rare benign neoplasms that pose diagnostic challenges. Complete resection is the optimal option for diagnostic confirmation and recurrence prevention. Laparoscopic surgery is feasible for children with mesocolic lymphangioma.

## Introduction

Cystic lymphangiomas (CLs) are uncommon, benign malformations of the lymphatic system (1), which primarily occur during childhood, more than 80% of cases appear before the age of two (2). Most are located in the head-neck region(75%), and axilla(20%), while rarely arise in the abdomen (3). Abdominal cystic lymphangiomas (ACLs) constitute less than 5% of all cystic lymphangiomas (4), with the most common location in the mesentery and retroperitoneum and exceptionally rare in the mesocolon (5).

The clinical feature varies from incidental findings on imaging to acute abdomen mimicking a variety of pathologies including appendicitis, pancreatitis, and even malignancies (6). Due to their low frequency and acute abdominal condition, preoperative diagnosis remains challenging (7).

Herein, we describe a case of mesocolic cystic lymphangioma presenting with recurrent dull abdominal pain, in which laparoscopic surgery was performed successfully. Moreover, we updated the clinical features of this rare disease through a literature review. To our knowledge, our review contains the largest case series of mesocolic cystic lymphangioma to date.

## Case presentation

**Figure 1:**
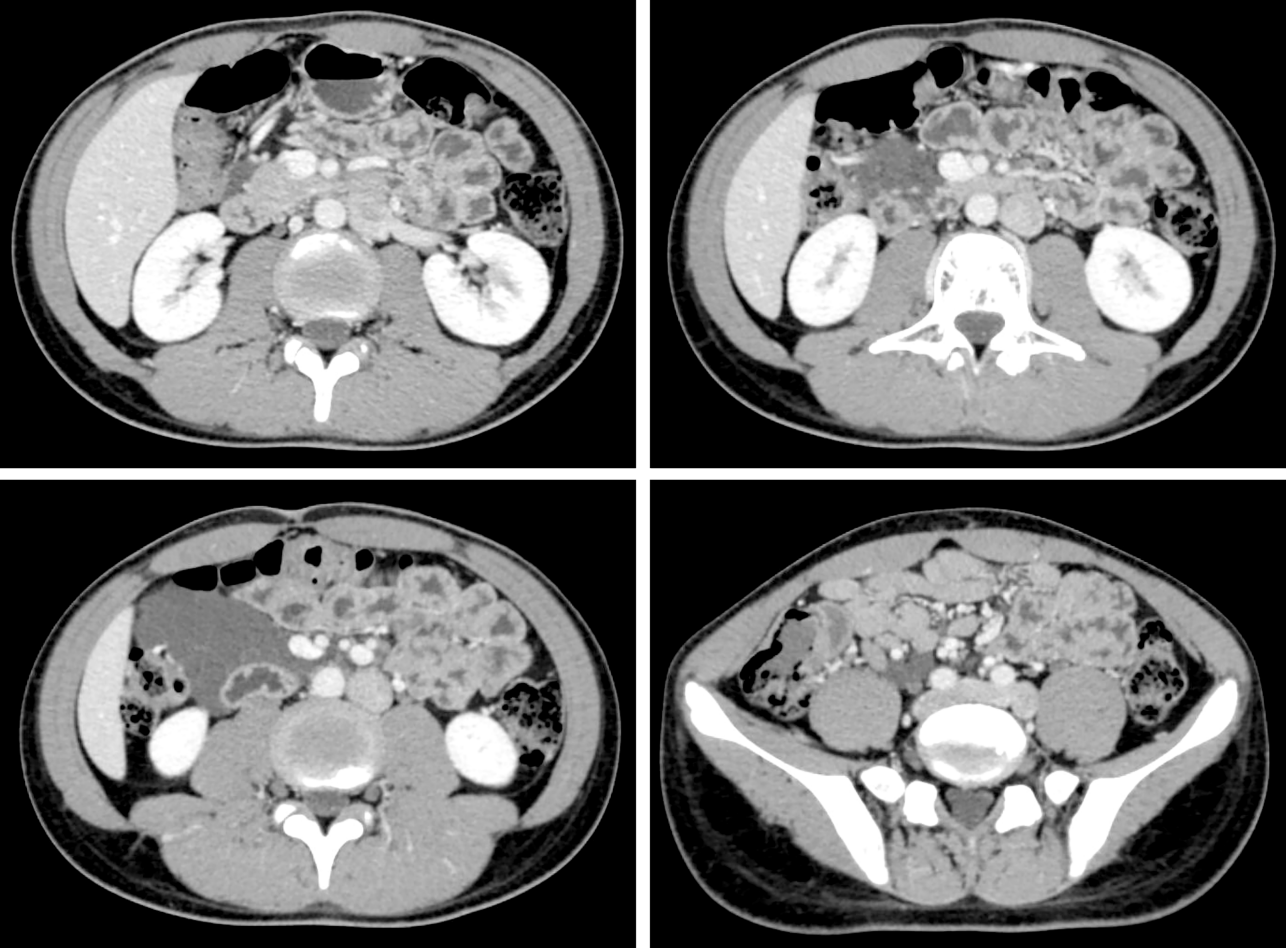
Computed tomography showed a large cystic mass.

This study was reported under the principle of the CARE guideline (8). On April 18, 2022, a 14-year-old male was referred to West China Hospital for further investigations regarding the diagnosis of an intraabdominal mass. He had been experiencing recurrent dull abdominal pain for 8 months, which became aggravated one month ago. Persistent pain concentrated around the umbilicus for 1 to 2 hours, with gradual relief after resting. Any concomitant symptom, including fever, chill, vomiting, nausea, melena, and diarrhea, was denied. The patient reported unremarkable past medical history and family medical history. Physical examination revealed a palpable mass extending from the epigastric to the hypogastric region, particularly on the right side of the umbilicus. Neither routine laboratory investigations nor tumor marker testing revealed any abnormalities (**Table 1**). Computed tomography (CT) showed a clumpy cystic low-density lesion on the right mesocolon (maximum cross-section,7.8×5.1cm). No enhancement was observed on contrast-enhanced CT, and the adjacent mesangial lymph nodes were slightly enlarged, suspecting a cyst lymphangioma (**Figure 1** **)**. To confirm the diagnosis and relieve symptoms, a laparoscopic operation was performed on April 25, 2022.

**Table 1:** Measurement of plasma tumor markers on April 22, 2022.

	Patient values	Units	Ref Range	Status
CEA	1.19	ng/mL	<5	–
CA199	<2.00	U/mL	<30	–
CA125	13.30	U/mL	<24	–
AFP	1.49	ng/mL	<7	–

Under general anesthesia, a laparoscopic procedure was performed by transperitoneal approach. The patient assumed a supine position. A Veress needle was used to establish pneumoperitoneum, maintaining a constant pressure at 13 mmHg. Two 12-mm trocars were respectively inserted on the right and left side of the abdomen, 5cm below the umbilicus. The operation was carried out using an ultrasonic surgical aspirator (CUSA; Cavitron Laser-sonic Corp., Stamford, Connecticut, USA), harmonic scalpel (Ethicon Endo-Surgery, Inc., Blue Ash, Cincinnati, OH, USA), and a bipolar clamp coagulation system (ERBE, Tubingen, Germany). A large cystic mass covered the right mesocolon was found intraoperatively. The upper margin of the lesion reached the descending part of the duodenum, and the lower margin reached the beginning of the right common iliac artery. The lesion was carefully isolated from the surrounding tissue and then stripped from the capsule through blunt dissection. Colonic resection was not done. Tumor was completely excised and the resection specimen was collected in a plastic bag and removed *via* a 5-cm subxiphoid incision. After ensuring that there was no active bleeding in the abdominal cavity, a drainage tube was placed in the retroperitoneal space. At the end of the procedure, instruments were counted, trocars were removed, pneumoperitoneum was evacuated, and the incisions were sutured. The operation took 135 minutes and the blood loss was estimated at 20ml.

**Figure 2:**
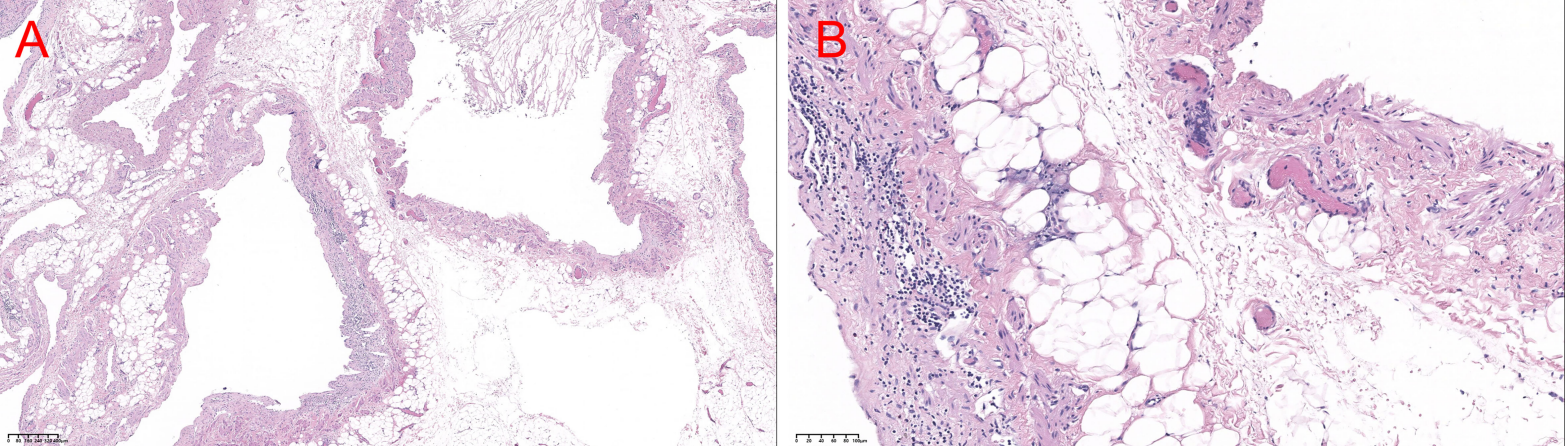
HE stains in **(A)** 40× view and **(B)** 200× view. Histology of lymphangioma. The cystic wall consisted of fibroconnective tissue accompanied by dilated lymphatic spaces and lymphoid cell aggregations in the endothelial lining of lymphatic vessels **(A)** H&E ×40, **(B)** H&E ×200.

Gross specimens showed that the mass was measured at 8×7cm with line-like septal shadow, irregular shapes, and mainly cystic components. The cyst wall was irregularly thickened with clear and transparent cyst fluid. Histological analysis confirmed the final diagnosis of mesocolic cystic lymphangioma (**Figure 2**).

The patient had no postoperative complications and abdominal pain symptoms disappeared dramatically after the procedure. He recovered uneventfully following the surgery and was discharged home on the fifth postoperative day. He and his family were satisfied with the outcome and receptive to the follow-up recommendations. No evidence of recurrence was found during the 3-month follow-up period.

## Discussion

Lymphangiomas are rare benign lesions that can be categorized as capillaries, cavernous, or cystic based on histological characteristics (3). Lymphatic system obstruction during embryological development causes congenital lymphangiomas, although the precise cause remains unclear. Several chromosomal abnormalities are linked to cystic lymphangiomas, including trisomies 13, 18, and 21, Noonan syndrome, Turner syndrome, and Down syndrome (9). In addition to the delayed proliferation of congenital tumors, acquired lymphangioma in response to stimuli, such as infection, local trauma, surgery, or radiation therapy may also explain the occurrence in the adult population (10). Our patient developed chronic abdominal pain for the first time after attaining an adolescent age, without any acquired etiology.

Cystic lymphangioma is diagnosed usually under the age of two (80%–90%) with an incidence of 1.2–2.8 per 100,000 children (11) and is even rarely seen during adulthood. It most generally occurs in the head, neck, and axillary region, due to the presence of an extensive lymphatic system. Fewer than 5% of lymphangiomas originate in the abdominal cavity, with the most common site being the small bowel mesentery and there are only 22 detailed reports of mesocolic lymphangiomas in the English literature (3, 5, 6, 12–28). A summary of the characteristics for all included reports is presented in **Table 2** Our study reports a case of mesocolic lymphangioma in a 14-year-old child and conducts a literature review. To our knowledge, this is the largest detailed case description of mesocolic lymphangioma successfully operated through the laparoscope. It provides the latest, along with the first tabular bibliography review of mesocolic lymphangioma with detailed clinical features, meanwhile.

**Table 2:** A summary of reported mesocolic lymphangioma, arranged by year.

First Author	Year	Country	Size (cm)	Sex	Age	Location	Histopathological evaluation	Operation approach	Hospital stays	Chief complaint	DiagNostic imaging	Follow-up	Recurrence
Current case	2022	ChiNR	8.0 × 7.0	M	15	right mesocolon	benign	laparoscopic	5 days	abdomiNRl pain	CT	3months	No
Tuan NR	2021	Viet NRm	15.2 × 16.8 × 3.2	F	15	right mesocolon	benign	Open	5 days	abdomiNRl pain	CT	3months	No
Abdulraheem AK	2021	Jordan	8.0 × 5.0 × 4.0	F	1-year 9-month-old	left mesocolon	benign	Open	5 days	abdomiNRl pain and fever	X-ray\US\CT	NR	NR
Guachilema Ribadeneira A	2020	Ecuador	11×8.2 ×7.6	F	60	sigmoid mesocolon	NR	Open	3 days	abdomiNRl pain	US\CT	NR	NR
Bang GA	2019	Cameroon	33 ×30×25	F	46	transverse mesocolon	benign	Open	5 days	abdomiNRl distension	CT	NR	NR
Hirata Y	2017	Japan	3	M	33	left mesocolon	NR	Open	15days	abdomiNRl pain and fever	CT	1 year	No
NRganuma H	2017	Japan	24×14	F	24	right mesocolon	benign	Open	NR	abdomiNRl pain	US\CT	1 year	No
NRganuma H	2017	Japan	13×10	M	55	sigmoid mesocolon	NR	Open	NR	abdomen pain and distention	US\CT	2 year	No
Shah A	2014	Ireland	6×4.5 ×2.5	F	33	mesocolon	NR	laparoscopic	5 days	abdomiNRl pain	CT	2year	No
Bhandarwar AH	2013	India	5 × 6	F	42	sigmoid mesocolon	benign	laparoscopic	1 days	abdomiNRl pain	US\CT	2 year	No
Kambakamba P	2012	Switzerland	34×17×25	NR	34	left mesocolon	benign	laparoscopic conversion laparotomy	5 days	asymptomatic	CT	1 year	No
Wang JH	2012	ChiNR	6×5×3.2	M	26	sigmoid mesocolon	NR	laparoscopic	4 days	abdomiNRl pain	MRI	NR	NR
Limdi JK	2010	UK	20×10	M	46	left mesocolon	NR	Open	NR	abdomiNRl pain and distension	US\CT	NR	NR
Ha TK	2009	Korea	25 × 15× 10	F	47	left mesocolon	NR	Open	NR	abdomiNRl pain	CT	NR	NR
Nizami S	2007	Pakistan	9×5×4	M	42	transverse mesocolon	benign	Open	5 days	abdomen pain and distention	US\CT	NR	NR
Güvenç BH	2005	Turkey	1.3×0.6	F	3	left mesocolon	NR	laparoscopic conversion laparotomy	NR	abdomiNRl distention and respiratory distress.	US\CT	NR	NR
Hauser H	1997	Austria	13×8×7	F	72	transverse mesocolon	benign	Open	NR	abdomiNRl pain	X-ray\CT	NR	NR
Iwabuchi A	1997	Japan	2 to 5 (maximum size, 10 )	M	31	dissemiNRted	NR	Open	NR	Severe anemia with gastrointestiNRl bleeding	CT\MRI	NR	NR
Kubota A	1996	Japan	NR	F	1	right mesocolon	NR	Open	NR	abdomen distention and fever	MRI	NR	NR
Mayer M	1994	German	NR	M	10	transverse mesocolon	NR	Open	NR	abdomiNRl pain	NR	NR	NR
Yuen ST	1992	ChiNR	NR	F	14	transverse mesocolon	NR	Open	NR	NR	NR	NR	NR
Nordshus T	1976	NR	NR	F	4	transverse mesocolon	NR	Open	NR	enlargement of the abdomen	US	NR	NR

NR:Not Reported

Mesocolic lymphangioma lacks distinct clinical signs and symptoms, and patients are often admitted to the hospital with complaints of abdominal pain (15 cases, 68.2%), followed by abdominal distension (6 cases, 27.3%). It appears that mesocolic lymphangiomas primarily involve the transverse colon (6 cases, 27.3%) or descending colon (6 cases, 27.3%). However, some caution should be exercised in interpreting this finding given the limited documentation of the case. Females (13 cases, 59.1%) are more likely to be suffered than males (8 cases, 36.4%). Bang GA et al. (12) reported the largest and heaviest mesocolic lymphangioma in 2019, which measured about 33 × 30 ×25 cm and weighed 16 kg. The long-term outcome of this disease is excellent, with no report of recurrence.

Tests in the laboratory are nonspecific but can be used to rule out malignant behavior of other etiology. Ultrasonography is of high diagnostic value in detecting mesenteric cystic lymphangioma (MCL) which usually exhibits a cystic mass accompanying multiple thin septations (13, 29). Considering its ability to determine the anatomical relations of the lesion, which ultimately guided treatment options and surgical approaches, CT scans remain the best radiological tool for evaluating MCL (mesenteric and mesocolon). In addition, CT provides clarity on size, density, and enhancement properties, which contributes to differential diagnosis (6, 29). Magnetic resonance imaging (MRI) shows better sensitivity for detecting the nature of cystic contents and intracavitary hemorrhages. Despite this, it is still challenging to make a preoperative diagnosis due to its rarity and absence of typical characteristics. Acute abdomen, cystic teratomas, tuberculosis, pseudocysts, enteric duplication cysts, ovarian tumors, other primary mesenteric tumors, and metastatic diseases should also be considered in the differential diagnosis (30, 31).

Conservative treatment and surgical excision are the two options once diagnosed. Sclerotherapy, which involves directly puncturing the cyst, aspirating the fluid, and injecting the sclerosing agents (SAs), has been applied for decreasing the mass size or regressing. A variety of SAs has been tested, including ethanol, sodium tetradecyl sulfate, and doxycycline; among them, bleomycin and picibanil (OK-432) are the most extensively utilized and studied. However, the long-term consequences of sclerotherapy are controversial (5, 32). Also, it has proven to be not very effective for microcystic disease (33). In addition, concerns should be raised about sclerotherapy complications including skin necrosis, local neuropathy, fibrosis or obliteration of lymphatic vessels, and, in rare cases, dose-dependent cardiopulmonary toxicity needs attention (34). Recently, lymphaticovenular anastomosis (LVA) combined with ethanol sclerotherapy has been reported to have a satisfactory outcome, suggesting that it could be a complementary minimally invasive treatment (35). However, further observation and cumulative cases are required to elucidate the accurate indications.

To confirm the diagnosis, relieve associated symptoms, and prevent any potential complications, surgical resection is suggested. All cases we reviewed in the present study were treated surgically. Since incomplete resection results in 10%-40% of recurrences of MCL (36–38), complete resection should also be indispensable for mesocolic lymphangioma. Depending on whether there is a pedicle, bowel segment resection may require for the tumor (39). In recent years, laparoscopic surgery, which could reduce postoperative discomfort and shorten hospital stay, has been successfully applied to excise mesocolic lymphangioma. To date, six cases (15–18, 22) of laparoscopic resection of mesocolic lymphangioma have been reported. Among them, two cases (17, 22) were converted to laparotomy. In this report, we describe the largest case smoothly managed by laparoscopy to date. However, one limitation of our case is the short follow-up period (3 months). A longer follow-up is required to observe the prognosis for this patient.

To sum up, we report a rare case of mesocolic lymphangioma onset in adolescence and performed an up-to-date literature review of patients with such neoplasm. Typically, patients present with acute or chronic abdominal pain. Imaging examination is helpful for preoperative diagnosis, and complete resection is recommended.

## Data availability statement

The original contributions presented in the study are included in the article/supplementary material. Further inquiries can be directed to the corresponding author.

## Ethics statement

Written informed consent was obtained from the individual(s) for the publication of any potentially identifiable images or data included in this article.

## Author contributions

XF, XC drafted and revised the manuscript. HL, XL, HC, and ZC collected data. JL and QF revised the manuscript for content. XF and JL designed the study and revised the manuscript. All authors contributed to the article and approved the submitted version.

## Funding

This work was supported by Sichuan University from 0 to 1 project (No. 2022SCUH0017); Sichuan Science and Technology Plan Project “International cooperation in science and technology innovation/technological innovation cooperation in Hong Kong, Macao, and Taiwan” (No. 2021YFH0095).

## Conflict of interest

The authors declare that the research was conducted in the absence of any commercial or financial relationships that could be construed as a potential conflict of interest.

## Publisher’s note

All claims expressed in this article are solely those of the authors and do not necessarily represent those of their affiliated organizations, or those of the publisher, the editors and the reviewers. Any product that may be evaluated in this article, or claim that may be made by its manufacturer, is not guaranteed or endorsed by the publisher.
